# “A mother somewhere between life and death”: a qualitative study of mothers diagnosed with bipolar disorder within a feminist social work framework

**DOI:** 10.3389/fpsyt.2026.1951897

**Published:** 2026-10-30

**Authors:** Selda Meydan, Emel Yurtsever, Mustafa Karataş

**Affiliations:** 1 Department of Social Work, School of Health Sciences, Istanbul Medipol University, Istanbul, Türkiye; 2 Department of Social Work, Faculty of Health Sciences, Istanbul Sabahattin Zaim University, Istanbul, Türkiye; 3 Department of Social Work, Faculty of Health Sciences, Kütahya Health Sciences University, Kütahya, Türkiye

**Keywords:** bipolar disorder, feminist social work, gender, motherhood, qualitative research

## Abstract

**Objective:**

To examine how women diagnosed with bipolar disorder experience and negotiate motherhood through a critical-feminist social work lens.

**Methods:**

This study conducted a supplementary qualitative secondary analysis within a qualitative design informed by a critical-feminist epistemological position. Semi-structured, in-depth interviews with eight unemployed mothers receiving treatment at the psychiatry clinic of a foundation university hospital in Türkiye were reanalyzed using reflexive thematic analysis. The analysis combined inductive coding with theoretically informed critical-feminist interpretation.

**Results:**

Six themes captured constrained maternal identity, interruptions to everyday caregiving, guilt and maternal self-evaluation, perceived fragility in mother–child relationships, motherhood as a source of meaning and strength, and shifting family support roles ranging from ordinary assistance to one explicit account of perceived role reversal. These accounts were interpreted in relation to gendered motherhood norms, family authority, and material constraints.

**Conclusion:**

The findings highlight the need for gender-sensitive, family-oriented psychosocial interventions for mothers diagnosed with bipolar disorder.

## 1 Introduction

Women’s experiences of mental illness are shaped by clinical symptoms, caregiving responsibilities, gender norms, family relationships, and structural conditions (1–4). For women living with bipolar disorder, episodic changes in mood and functioning may intersect with expectations of continuous maternal availability, influencing everyday caregiving and maternal identity. Bipolar disorder is characterized by episodes of mania, hypomania, and depression that can substantially affect mood, energy, and functioning (5–7) and impair social, occupational, and interpersonal functioning (8–10). In 2023, approximately 36 million people, or 0.4% of the global population, were living with bipolar disorder (11). Cross-national lifetime prevalence estimates are 0.6% for bipolar I disorder, 0.4% for bipolar II disorder, and 2.4% for the broader bipolar spectrum (12).

Beyond symptom management, health social work addresses psychosocial functioning, family relationships, social support, and access to community resources (13–15). These dimensions are particularly relevant to women, who disproportionately undertake unpaid domestic and caregiving labor while encountering gender inequalities and mental illness-related stigma (16–19). Feminist social work situates women’s mental health within gendered expectations, power relations, and social inequalities while emphasizing empowerment and social justice (20–22). An intersectional perspective further recognizes that gender interacts with social class, employment, education, psychiatric status, and family power relations, shaping access to income, childcare, support, and decision-making authority (3, 23, 24).

In Türkiye, motherhood is often constructed through expectations of sacrifice, continuous emotional availability, and intensive maternal responsibility (25–27). Illness-related disruptions may conflict with these expectations and contribute to guilt, inadequacy, and self-questioning. Mental health care is delivered through hospital-based psychiatric services and community mental health centers, which provide treatment monitoring, psychosocial rehabilitation, home visits, and patient and family education (28, 29). Social workers contribute through psychosocial assessment, counseling, family work, resource linkage, and service coordination (30). However, a standardized parenting-focused service for parents with severe mental illness has not been identified within this framework.

Qualitative evidence on parenting with bipolar disorder has recently been synthesized. Tu et al. (31) reviewed 19 studies and identified four broad domains: the effects of mood fluctuations on parenting, changing parent–child relationships, the dual emotional meaning of childcare, and strategies for navigating parenting challenges. Tu et al. (32) further examined communication about parental bipolar disorder with children. However, this literature is predominantly Western, and less attention has been given to how these experiences are shaped by gendered motherhood expectations, socioeconomic positioning, and family power relations within critical-feminist social work. In Türkiye, research has largely addressed stigma, social support, reproductive experiences, and clinical processes, while the intersection of bipolar disorder, maternal identity, caregiving expectations, and family relationships remains underexplored (33–36). The contribution of the present study therefore lies not in claiming previously unknown parenting difficulties but in providing a contextually situated interpretation of how they are experienced and negotiated by mothers receiving clinical care in Türkiye.

This study examines how women diagnosed with bipolar disorder experience and negotiate motherhood at the intersection of bipolar disorder, gendered caregiving expectations, and family relationships. Through a critical-feminist social work interpretation, it considers how gender norms, family power relations, and structural inequalities shape these accounts and their implications for gender-responsive, family-oriented psychosocial practice.

## 2 Materials and methods

### 2.1 Research design

This study used a qualitative design informed by a critical-feminist epistemological position and conducted a supplementary qualitative secondary analysis of existing interview data. Participants’ accounts were treated as situated forms of meaning-making shaped by gendered power relations, caregiving norms, family relationships, and structural inequalities, while recognizing the researchers’ active interpretive role (20, 37). Qualitative secondary analysis enables existing data to be revisited for a new or more focused research question when they provide sufficient contextual and conceptual fit (38, 39). Within Heaton’s (40) typology, this study constitutes a supplementary analysis because it examines motherhood-related material that was generated in the parent study but not fully developed in the original analysis.

The parent doctoral study included 15 women diagnosed with bipolar disorder and examined their daily lives, social roles, stigma, and gendered experiences. Although motherhood-related accounts were considered in the initial analysis, they were not examined through a mother-only cross-case analysis. Interviews with all eight mothers contained detailed accounts of caregiving disruptions, maternal guilt and identity, family responses, role redistribution, and the meanings attributed to motherhood; the metaphor-based question generated further conceptually rich material. The complete interviews of these eight mothers were therefore recoded and reinterpreted through a feminist social work lens to examine how motherhood is experienced and negotiated at the intersection of bipolar disorder, gendered caregiving expectations, and family relationships, rather than merely extracting motherhood-related findings from the original analysis.

### 2.2 Participants

Of the 15 women in the parent doctoral study, eight were mothers, and all eight were included in the present secondary analysis. This mother-only subgroup constituted a purposive sample directly relevant to the focused research question (41, 42). Because the dataset was closed, sample size was determined by the number of mothers in the parent study rather than by prospective recruitment or data saturation. Sample adequacy was assessed through information power, considering the specificity of the subgroup, the focused research question, the approximately 70-minute interviews, and the richness of the motherhood-related accounts (37, 43).

Diagnoses and diagnostic subtypes were verified from clinical records: five participants had bipolar I disorder and three had bipolar II disorder. Episode histories, hospitalizations, and treatment adherence were based on participants’ self-reports. Participants described histories of manic or hypomanic and depressive episodes, with depressive experiences predominating. The reported frequency and duration of mood changes varied; some participants retrospectively described periods occurring approximately monthly and lasting around two weeks. However, these accounts were not verified against longitudinal clinical records or standardized diagnostic assessments, and the study could not determine whether they constituted distinct mood episodes or met the criteria for a rapid-cycling course. Each participant reported at least two psychiatric hospitalizations, and one had a comorbid diagnosis of borderline personality disorder.

At the time of the interviews, four participants were receiving both pharmacotherapy and psychotherapy, two pharmacotherapy alone, and two psychotherapy without current pharmacotherapy. Six reported regular treatment adherence, whereas two reported irregular adherence. Both participants without current pharmacotherapy had previously used medication. One had recently given birth and intended to resume pharmacotherapy, whereas the other had temporarily discontinued medication because of substantial side effects. These categories therefore reflect treatment status at the time of the interviews rather than stable or long-term treatment patterns. Interviews were conducted when participants reported no acute mood episode and felt sufficiently well to participate. Because mood state was not assessed using a standardized measure, formal remission or euthymia could not be established.

Eligibility for the parent study required participants to be aged 18 years or older, have a psychiatrist-confirmed diagnosis of bipolar disorder, provide written informed consent, and be sufficiently well to participate in an interview and express themselves. Individuals experiencing active psychosis were excluded. Motherhood experience was the additional eligibility criterion for the present supplementary analysis. Participants’ sociodemographic and family characteristics are presented in **Table 1**.

**Table 1:** Sociodemographic and family characteristics of participants.

Participant	Age	Marital status	Educational level	Employment status	Number of children	Age(s) of children	Child(ren) co-residing with the mother	Custody/care arrangement	Duration since diagnosis
P1	39	Divorced	Primary school graduate	Unemployed	1	17	No (lives with father)	Sole paternal custody	23 years
P2	35	Married	Middle school graduate	Unemployed	1	10	Yes	Not applicable (parents married)	17 years
P3	43	Divorced	Primary school graduate	Unemployed	1	18	No (lives with father)	Not applicable (adult child)	10 years
P4	40	Married	Primary school graduate	Unemployed	2	16, 18	Yes (all)	Not applicable (parents married)	2 years
P10	33	Married	Middle school graduate	Unemployed	4	1, 3, 8, 12	Yes (all)	Not applicable (parents married)	13 years
P11	31	Married	High school graduate	Unemployed	1	5	Yes	Not applicable (parents married)	7 years
P12	37	Married	University graduate	Unemployed	1	14	Yes	Not applicable (parents married)	4 years
P15	53	Married	Primary school graduate	Unemployed	2	24, 30	Yes (all)	Not applicable (adult children)	33 years

Children’s ages and living arrangements refer to the time of the interviews. Duration since diagnosis was based on participants’ self-reports. Participant codes were used to protect confidentiality.

### 2.3 Data collection instruments

Data were collected in the parent study using a sociodemographic information form and a semi-structured interview guide. The sociodemographic form recorded participants’ age, marital status, educational level, employment status, duration since diagnosis, and information concerning their children and caregiving arrangements. The interview guide was developed by the first author based on the relevant literature and included open-ended questions addressing participants’ experiences of diagnosis, daily life, social roles, stigma, gender, and motherhood. Its flexible structure allowed participants to elaborate on issues they considered important.

The parent study also included a metaphor-elicitation question designed to explore the meanings participants attributed to motherhood: “A child (son/daughter) is like … because ….” Both the metaphor and its explanatory “because” component were examined across participants and interpreted alongside the relevant interview narratives. The metaphor material contributed to the development of the theme “Motherhood as a source of meaning and strength” rather than constituting a separate theme.

### 2.4 Data collection process

Data for the parent study were collected between March 2022 and May 2023. Potential participants were initially approached by their treating psychiatrist. The first author subsequently provided written and verbal information about the study and obtained written informed consent. Participants were informed that participation was voluntary, that they could refuse or withdraw without providing a reason, and that their decision would not affect their treatment or access to services. The researcher had no role in participants’ treatment or clinical decision-making, and the research interview was explicitly presented as separate from clinical care to minimize therapeutic misconception and implicit pressure.

All interviews were conducted by the first author, who had no prior relationship with the participants. At the time of data collection, she was a female social work academic and doctoral student, held a master’s degree, and had received doctoral-level training in qualitative research. Participants were informed about her professional role, research interests, and the purpose of the doctoral study. Interviews were conducted face-to-face in a private room at the hospital, lasted approximately 70 minutes, and were audio-recorded with participants’ consent. No one other than the participant and interviewer was present. Interview appointments were arranged individually according to participants’ availability and reported clinical stability. Each participant was interviewed once, and field notes were prepared for each interview. Transcripts were not returned to participants for comment or correction.

### 2.5 Data analysis

The interview transcripts were reanalyzed using reflexive thematic analysis within a critical-feminist epistemological orientation (37). Themes were understood as researcher-produced patterns of shared meaning developed from situated accounts rather than as entities that simply emerged from the data.

Following Braun and Clarke’s six recursive phases, the first author repeatedly read the complete transcripts of all eight mothers and coded both direct responses to motherhood questions and relevant material elsewhere in the interviews. Codes were compared across cases and organized around central concepts, and candidate themes were reviewed against the coded extracts, complete dataset, and research question. MAXQDA 2024 was used to organize the data, not to generate themes or interpretations.

Coding was initially inductive and close to participants’ accounts, while feminist social work and intersectionality served as sensitizing perspectives rather than a predetermined coding framework (20, 23). The analysis examined caregiving responsibility and decision-making authority, the interpretation of illness- and treatment-related disruptions as maternal inadequacy, and how employment, education, and access to paid or family care shaped participants’ options. It also examined contradictions, including family involvement as both support and restriction, motherhood as both constraint and source of meaning, and children’s assistance as ordinary support or perceived role reversal.

Extracts were selected to illustrate convergence, variation, and contradiction. Accounts were compared across age, duration since diagnosis, marital and custody status, number and ages of children, co-residence, education, and employment. Deviant or qualifying cases were used to contextualize within-sample variation rather than infer subgroup differences. Because most participants did not explicitly frame their experiences in terms of gender, gendered meanings were treated as theoretically informed interpretations. The absence of a comparison group precluded claims that the reported difficulties were specific to mothers rather than primary caregivers more generally. The first author’s social work background and interest in women’s mental health were treated reflexively as part of the analytical process.

All interviews were conducted, transcribed, and analyzed in Turkish. The manuscript and participant extracts were translated into English with the assistance of ChatGPT (OpenAI) and checked against the original Turkish texts by a co-author proficient in both Turkish and English. Idiomatic expressions were translated for conceptual rather than literal equivalence; no formal back-translation was conducted (44).

### 2.6 Methodological rigor and analytical quality

Methodological rigor was supported by alignment among the focused research question, critical-feminist epistemological orientation, supplementary secondary analysis design, and reflexive thematic analysis. Before data collection, the literature-informed interview guide was reviewed by multidisciplinary mental health experts and five women who subsequently participated in the parent study. Their feedback led to the merging of overlapping or overly detailed questions and the simplification of wording. This feedback concerned the interview guide, not the themes or study findings.

Analytical quality was supported through sustained engagement with the complete dataset, recursive movement between coding and theme development, reflexive note-taking, and documentation of changes to the thematic structure. The first author led the analysis, while the co-authors served as critical dialogue partners. When interpretations differed, the authors revisited relevant extracts and considered alternative readings. Consistent with reflexive thematic analysis, the purpose was critical reflection rather than coding consensus, and the first author retained responsibility for final analytical decisions (37). Quotations were used to make the relationship between participants’ accounts and the interpretations transparent. The manuscript was reviewed against COREQ; the completed checklist and original interview guide are provided as **Supplementary Materials 1**, **2** (45).

## 3 Results

The reflexive thematic analysis generated six themes that capture how participants negotiated maternal identity, caregiving responsibilities, and family relationships in the context of bipolar disorder. The themes were: (1) negotiating maternal identity under illness and treatment constraints; (2) when illness episodes interrupt everyday caregiving; (3) internalizing responsibility: guilt and maternal self-evaluation; (4) perceived fragility in the mother-child relationship; (5) motherhood as a source of meaning and strength; and (6) shifting care across generations: support and perceived role reversal. Together, these themes show that illness- and treatment-related disruptions were experienced alongside guilt, relational uncertainty, changes in family support, and motherhood’s continuing role as a source of attachment and meaning. The thematic structure is summarized in **Table 2**.

**Table 2:** Thematic structure, underlying codes, participant coverage, and representative extracts.

Theme	Underlying codes	Participant coverage	Representative extract
Negotiating maternal identity under illness and treatment constraints	Emotional and depressive withdrawal; medication-related sedation; reduced physical and emotional availability; social questioning of maternal competence; expectations of continuous maternal presence	3/8 (P1, P11, P12)	“You become a mother who is constantly ill and bedridden—a mother somewhere between life and death.” (P12)
When illness episodes interrupt everyday caregiving	Medication-related interruption of breastfeeding; inability to meet daily care needs; disruption of household routines; need for paid caregiving support	4/8 (P2, P3, P12, P15)	“I had to stop breastfeeding because I was taking medication.” (P2)
Internalizing responsibility: guilt and maternal self-evaluation	Maternal guilt and inadequacy; compensatory parenting; pregnancy–treatment dilemmas; perceived hereditary risk; anticipated effects of future episodes on children	5/8 (P2, P4, P10, P12, P15)	“During depressive periods, I think that I am inadequate.” (P10)
Perceived fragility in the mother-child relationship	Restricted caregiving opportunities; loss of maternal authority; perceived emotional distance; self-attributed neglect; fears of burdening or harming children	4/8 (P2, P10, P12, P15)	“My relationship with my son was negatively affected as well.” (P12)
Motherhood as a source of meaning and strength	Attachment and devotion; active and attentive parenting; perceived maternal competence; children as sources of recovery, strength, warmth, and hope	Narrative accounts: 3/8 (P2, P10, P11); metaphor responses: 8/8 (all participants)	“My child helps me get back on my feet.” (P10)
Shifting care across generations: support and perceived role reversal	Household assistance; instrumental and emotional support; awareness of the mother’s illness; emotional monitoring; perceived role reversal and rescue	4/8 (P3, P4, P10, P15)	“My children became my parents … They always acted like my rescuers.” (P15)

Participant coverage indicates the number of participants whose accounts contributed to each theme. The figures are descriptive and do not indicate prevalence or thematic importance. For the theme “Motherhood as a source of meaning and strength, “ narrative and metaphor-based coverage are reported separately. Five participants (P1, P3, P4, P12, and P15) contributed to this theme only through their metaphor responses.

### 3.1 Negotiating maternal identity under illness and treatment constraints

My daughter was born after I received the diagnosis. For example, I did not want to hold my daughter in my arms. (P1)

I think being a mother, being a woman, and living with bipolar disorder in this country is extremely difficult. For example, I had a young child at home. She was only two years old, and I needed to take care of her, but I was taking very heavy medications and staying in bed 24/7. I could not even lift my head up; it felt as if I did not exist. (P11)

During the depressive period, I could not take care of my son at all. The illness keeps you outside of life; it limits you in many ways. After becoming ill, there was a mother who slept for an entire month. You become a mother who is constantly ill and bedridden—a mother somewhere between life and death. (P12)

For example, people close to me made comments such as, “How will she take care of a child? How can she be a mother?” (P11)

The accounts illustrate how reduced physical and emotional availability—described in relation to diagnosis, depressive symptoms, and medication-related sedation—challenged participants’ sense of themselves as consistently present mothers. P11’s second account additionally illustrates how illness-related social judgments cast maternal competence into doubt, adding stigma to the practical constraints associated with symptoms and treatment.

### 3.2 When illness episodes interrupt everyday caregiving

During a period when I was experiencing an episode, I had to stop breastfeeding because I was taking medication. (P2)

Having children becomes a challenge for us. I mean, there may not be a problem with having children, but difficulties can arise during the caregiving stage. For example, during episode periods, I would need to hire a caregiver. (P3)

It affects women’s roles a lot as well. Providing care for your family becomes very difficult … You end up having the image of a mother who is constantly lying in bed. There is no food prepared at home. (P12)

When I took my medication, I could not get up. I could not prepare breakfast in the mornings. I was constantly sleeping. (P15)

Together, these accounts show how mood episodes and treatment disrupted routine caregiving tasks, including breastfeeding, preparing meals, and arranging childcare. Within the critical-feminist interpretation, these disruptions were experienced against ideals of continuous maternal availability; however, the findings do not establish that they were caused by gender or were unique to mothers.

P3’s reference to hiring a caregiver suggests that continuity of care during illness episodes may depend on access to paid support. Although unemployment and educational level do not establish poverty, all participants were unemployed and seven had secondary education or below, potentially limiting economic autonomy and access to paid childcare while increasing reliance on family support. Caregiving disruptions were therefore interpreted in relation to unequal material and relational resources as well as clinical symptoms.

### 3.3 Internalizing responsibility: guilt and maternal self-evaluation

During depressive periods, I think that I am inadequate. Afterwards, I try to make up for it, but how much can I really make up for? (P10)

I felt sad for my children. I kept thinking, ‘Am I a good enough mother? Can I meet their needs? Or am I neglecting them because of my illness?’ I felt inferior toward my children because of my illness. (P15)

These accounts show how participants interpreted illness-related caregiving difficulties as evidence of maternal inadequacy. Within the critical-feminist analysis, these self-evaluations were understood as expressions of guilt and internalized maternal accountability rather than as objective evidence of inadequate parenting. They illustrate how mother-blaming norms may lead women to personalize difficulties shaped by illness, treatment, and available support.

Motherhood is very different when you are living with bipolar disorder. In this illness, even having children becomes a major issue. You must stop your medication, but if you stop, the illness relapses, and an episode occurs. It feels like a dead end for us either way. Will I experience depression during pregnancy? Will I experience postpartum depression after the baby is born? Everything feels uncertain. Finding the courage to have another child is difficult… (P12)

Because this illness is genetic, my greatest concern is that my son may become ill like me. (P2)

I began to worry, “What if my children also develop bipolar disorder one day?” For a period, I kept thinking, “How might my episodes affect them? How might they be affected by this situation?” (P4)

Internalized maternal responsibility also extended into the future through concerns about pregnancy, treatment decisions, perceived hereditary risk, and the possible effects of future episodes on children. These accounts represent participants’ understandings and perceived dilemmas; they should not be interpreted as evidence of deterministic transmission or as clinical guidance regarding medication discontinuation.

### 3.4 Perceived fragility in the mother–child relationship

My son was eight months old when my mother-in-law took his crib from my room and moved it to hers. Afterwards, they did not give me the opportunity to care for my child. If they had supported me a little more, I could have taken care of my child myself, but they did not allow that. I missed out on so much time with my child. Even so, while raising him, I tried to do everything I could, but it was difficult. (P2)

P2’s account illustrates how family members’ assumption of caregiving responsibilities could restrict maternal decision-making and reduce opportunities for mother–child care. Family involvement was therefore interpreted as potentially supportive or disempowering depending on whether it strengthened maternal agency or displaced her authority within caregiving relationships.

No matter how much you expect them to understand, children are affected in the end. That is why I can say that my relationship with my son was negatively affected as well. This situation caused a great deal of harm to my child. (P12)

Because of this illness, I neglected my children a lot at certain points in time. I always felt sadness, thinking things like, ‘What have I done?’ But it was beyond my control. Of course, this also caused emotional distress in my children. (P15)

I think my children may have become tired of me or exhausted because of me. Maybe they would be better off without me. They are children after all; I do not want to place this burden on them. (P10)

These accounts convey participants’ perceptions that illness-related caregiving disruptions had created emotional distance and strained their relationships with their children. Because the children were not interviewed, these findings represent mothers’ meaning-making rather than independently verified developmental or psychological effects.

### 3.5 Motherhood as a source of meaning and strength

Motherhood is a very beautiful feeling. I think being a mother is what I have loved most in my life. Before my child woke up, I would wake up first to check on them and get up to breastfeed. (P2)

Even though I am ill, I am the parent who is most involved with my children. (P10)

In some respects, I think I am a more attentive parent than parents who do not have bipolar disorder. For example, I talk with my daughter’s teacher about her emotional well-being and behavior and try to notice any sudden changes or difficulties. (P11)

These accounts counter a uniformly deficit-oriented interpretation of motherhood. Participants described attachment, active involvement, and, in P11’s case, heightened attentiveness to a child’s psychological well-being, positioning motherhood as a domain of competence, meaning, and strength. P11’s comparison was interpreted as a subjective strength-based self-appraisal rather than as evidence that parents with bipolar disorder are generally more attentive than other parents.

The complete metaphor responses are presented in **Table 3**. “Angel,” “heart,” “cub,” and “gift” conveyed affection, closeness, and value, whereas “lioness,” “strength,” and “sun” emphasized strength, recovery, warmth, and hope. Read alongside the interview narratives, these metaphors reinforced the interpretation of motherhood as both vulnerable and sustaining rather than constituting a separate theme.

**Table 3:** Metaphors used by participants to describe their children.

Participant	Metaphor	Explanation
P1	Angel	“I see my child as an angel who was sent to me.”
P2	Heart	“My child is with me every moment.”
P3	Lioness	“She is very strong and capable of achieving what she wants.”
P4	Cub	“I love them very much.”
P10	Strength	“My child helps me get back on my feet.”
P11	Angel	“I truly see my child as an angel.”
P12	Sun	“My child warms and brightens me like the sun and makes me happy, just as flowers turn toward the sun and are warmed by it.”
P15	Gift	“I overcame many hardships for them, and I am glad that I brought them into the world.”

### 3.6 Shifting care across generations: support and perceived role reversal

My daughter also supports me with my illness as much as she can. (P3)

My younger daughter helps with household chores. (P4)

At this stage, my daughters are a huge source of support. They are very aware of my condition. (P10)

These accounts describe household assistance, emotional awareness, and general support, but do not in themselves demonstrate developmentally inappropriate caregiving. P4’s younger daughter was 16 at the time of the interview; her contribution to household chores may therefore reflect age-appropriate family collaboration rather than parentification.

My children became my parents. They thought things like, “I need to do something; I need to save my mother. I need to keep checking on her, so nothing happens.” They always acted like my rescuers. (P15)

P15, by contrast, explicitly described perceived role reversal and emotional monitoring. Because the timing, duration, and developmental context were not established and the children were not interviewed, this account was treated as the mother’s perception of shifting family roles rather than evidence of sustained parentification or harm.

Across all six themes, cross-case comparison suggested variation according to the children’s life stage. Accounts concerning infancy or early childhood foregrounded disruptions in hands-on care (P2, P10, and P11), whereas instrumental or emotional support was described mainly in accounts involving adolescent or adult children (P3, P4, and P15); P10 was a contrasting case, also describing support from her younger daughters. Only P15 explicitly described perceived role reversal. This pattern should be interpreted cautiously because **Table 1** records children’s ages at interview rather than at the time of every event. No consistent pattern was identified by maternal age, duration since diagnosis, or number of children, and the data did not support comparisons by marital or custody status. Overall, caregiving disruptions coexisted with attachment, active mothering, and changing forms of family support rather than forming a uniform deficit trajectory.

## 4 Discussion

Participants described depressive episodes and treatment-related effects as reducing physical and emotional availability and disrupting caregiving, consistent with qualitative research on parenting with bipolar disorder (31, 46, 47). These disruptions were often interpreted as personal maternal failures. The critical-feminist analysis situates this self-blame within ideals of continuous, self-sacrificing motherhood without claiming a gender-specific causal process (48). Concerns about pregnancy, treatment decisions, hereditary risk, and future episodes further extended maternal responsibility into anticipated futures. Although the clinical complexity of managing bipolar disorder during pregnancy and the postpartum period is well documented (49–51), the present findings show how such uncertainty may be experienced as a personal moral dilemma. Participants’ understandings should not, however, be interpreted as etiological evidence or clinical guidance.

Participants perceived illness-related disruptions as straining mother–child relationships, consistent with previous qualitative and clinical literature (31, 52). Because the children were not interviewed, these accounts represent mothers’ perceived relational consequences rather than independently established effects. Children’s involvement ranged from household assistance and emotional support to P15’s explicit account of perceived role reversal. Although parentification and shifting family roles have been documented among children of parents with mental illness (53, 54), this study did not establish the timing, duration, or developmental appropriateness of these responsibilities. Cross-case comparison suggested that hands-on disruptions were more prominent in accounts involving younger children, whereas support was more visible in those involving older children; however, retrospective timing prevented firm conclusions about life-stage differences. These findings therefore indicate changing family support roles rather than sustained parentification or harm.

Motherhood was also described as a source of attachment, meaning, competence, and recovery motivation, countering a uniformly deficit-focused interpretation (46, 47). The metaphor responses reinforced this interpretation through images of closeness, value, strength, warmth, hope, and recovery. Together, these accounts show that caregiving difficulties coexisted with active mothering and motherhood’s sustaining role.

The findings largely confirm the domains identified in Tu et al.’s (31) meta-synthesis—caregiving disruption, changing parent–child relationships, the dual meaning of childcare, and difficulties navigating parenting—but show how these patterns were configured within a Turkish clinical setting. P2 described extended family members assuming infant care while restricting maternal authority, consistent with Turkish research on relatives’ involvement during treatment and hospitalization (55), whereas P11 described psychiatric stigma through others’ questioning of her maternal competence. Unlike Tu et al. (32), which focused on communication with children, the present analysis examined maternal identity, caregiving responsibility, family authority, and socioeconomic constraints through a critical-feminist social work lens. Its contribution therefore lies in a locally situated interpretation of established patterns rather than in claiming entirely new or uniquely Turkish experiences.

Gender-related interpretations nevertheless require caution. Apart from P11’s explicit reference to being a mother and a woman living with bipolar disorder in Türkiye, most participants did not directly frame their difficulties as gendered. These meanings were developed through a critical-feminist reading of caregiving responsibility, maternal self-blame, and family authority. Without comparison groups of fathers diagnosed with bipolar disorder or mothers without the diagnosis, the study cannot distinguish the effects of gender, primary caregiving, bipolar disorder, or their interaction. An intersectional feminist social work perspective nonetheless prevents participants’ difficulties from being reduced to individual inadequacy or clinical symptoms alone (20, 23). All participants were unemployed, and seven had secondary education or below. Although these characteristics do not establish poverty, they suggest potentially constrained economic autonomy. Access to paid childcare and reliance on extended family shaped participants’ options, situating maternal distress within motherhood norms, unequal material resources, unpaid caregiving labor, and family decision-making power (3, 56).

## 5 Conclusion

This study shows that motherhood in the context of bipolar disorder involved caregiving disruptions, maternal guilt, relational concerns, and changes in family support, while also providing attachment, meaning, and motivation for recovery. The critical-feminist analysis situated these experiences within motherhood norms, material constraints, family authority, and psychiatric stigma without claiming gender-specific causation. Children’s involvement ranged from ordinary assistance to one explicit account of perceived role reversal, but the data do not establish sustained parentification or harm.

The findings support integrating gender-responsive, family-oriented psychosocial care with clinical treatment, including parenting-focused psychoeducation and family support that preserves maternal agency. Longitudinal and multi-informant research should examine how caregiving roles and relationships change over time and across sociocultural contexts.

## 6 Limitations

This study involved a secondary analysis of interviews with eight mothers receiving treatment at a single foundation university hospital, limiting transferability to other clinical and sociocultural contexts. Although the interviews contained rich motherhood-related accounts, they were not originally conducted solely for this focused research question. The findings also rely on retrospective self-reports. Episode histories and treatment adherence were not independently verified, and mood state at interview was not assessed using a standardized measure. Despite safeguards, recruitment within a treatment setting may also have created perceived pressure or therapeutic misconception.

The study included neither fathers diagnosed with bipolar disorder nor mothers without the diagnosis. It therefore cannot distinguish whether the reported difficulties reflected gender, primary caregiving, bipolar disorder, or their interaction. Apart from P11’s explicit statement, gendered meanings were developed through critical-feminist interpretation and should not be treated as evidence of gender differences.

Detailed data on household and personal income, housing security, social benefits, spouses’ employment, and financial decision-making were unavailable; consequently, poverty, social class, and economic dependency could not be directly examined. Data concerning the children were obtained only from mothers, and the children’s ages at interview may not reflect their ages when the reported events occurred. The findings therefore cannot establish psychological harm or sustained, developmentally inappropriate parentification. Future dyadic or multi-informant studies should include children’s perspectives and collect more detailed information on family resources and caregiving arrangements.

## Data Availability

The datasets presented in this article are not readily available because due to the sensitive and potentially identifiable nature of the interviews, raw transcripts are not publicly available, and participant consent did not cover the sharing of complete transcripts. De-identified excerpts supporting the analysis are presented in the article. Requests for additional de-identified materials may be considered only when compatible with the original consent and subject to ethics approval and applicable data-protection requirements. Complete raw transcripts will not be shared. Requests to access the datasets should be directed to selda.meydan@gmail.com.
