# Supplementary material for: “A mother somewhere between life and death”: a qualitative study of mothers diagnosed with bipolar disorder within a feminist social work framework
Source: Front Psychiatry. 2026 Oct 30;17:1951897. doi: 10.3389/fpsyt.2026.1951897 (PMC13759800; doi:10.3389/fpsyt.2026.1951897)
Supplement: Supplementary file 1 [file DataSheet1.pdf]

Supplementary Material 2
Semi-Structured Interview Guide Used in the Parent Doctoral Study

The following core questions guided the interviews. Depending on participants' responses, additional probing questions were used to help them elaborate on their experiences. The probes were not necessarily asked of every participant in the same order or using identical wording.

Questions Asked of All Participants

1. Could you tell me about the process of being diagnosed with bipolar disorder and what this diagnosis means to you?
2. How does bipolar disorder affect your daily life and the responsibilities you undertake? **Possible probes:** Mood episodes, daily routines, household responsibilities, working life, and social relationships.
3. How does the treatment process affect your daily life? **Possible probes:** Effects of medication, psychotherapy, access to treatment, and experiences of continuing treatment.
4. Have the attitudes of your family members or social environment toward you changed since you received the diagnosis? If so, how have you experienced these changes?
5. As a woman diagnosed with bipolar disorder, how do you experience the roles and expectations that society places on women? **Possible probes:** Being a spouse or mother, household responsibilities, caregiving, and social judgments.
6. Which people or resources support you in coping with difficulties associated with the illness? Are there forms of support that you need but have been unable to access?

Additional Questions Asked of Participants Who Were Mothers

7. What does being a mother mean to you? How do you think your experience of motherhood and your experience of bipolar disorder relate to one another?
8. How do mood episodes and the treatment process affect your ability to care for your child or children and your relationship with them? **Possible probes:** Daily caregiving, physical closeness, communication, time spent together, and periods when caregiving was interrupted.
9. What kinds of feelings and thoughts do you experience regarding your motherhood in relation to the illness? **Possible probes:** Feelings of adequacy, responsibility, guilt, anxiety, hope, and empowerment.
10. How are childcare responsibilities shared within your family? During periods of illness, do the responsibilities undertaken by family members or children change?
11. In what ways does motherhood provide a source of meaning or strength in coping with the illness?
12. Could you complete the following statement based on your own experience? **"A child (son/daughter) is like … because …."**

Closing Question

13. Is there anything else about your experience of living with bipolar disorder or motherhood that we have not discussed but that you would like to add?
