# Supplementary material for: “A mother somewhere between life and death”: a qualitative study of mothers diagnosed with bipolar disorder within a feminist social work framework
Source: Front Psychiatry. 2026 Oct 30;17:1951897. doi: 10.3389/fpsyt.2026.1951897 (PMC13759800; doi:10.3389/fpsyt.2026.1951897)
Supplement: Supplementary file 2 [file DataSheet2.pdf]

Supplementary Material 1: COREQ Checklist

Manuscript title: *"A mother somewhere between life and death": A qualitative study of mothers diagnosed with bipolar disorder within a feminist social work framework*

Page numbers refer to the Main manuscript-R1 dated 17 September 2026.

No.	Item	COREQ criterion	Reported in the manuscript
**Domain 1**	**Research team and reflexivity**		
1	**Interviewer or facilitator**	Identify the author who conducted the interviews or focus groups.	Reported. The first author conducted all interviews. Section 2.4, p. 6.
2	**Credentials**	State the interviewer's academic or professional qualifications.	Reported. The interviewer held a master's degree. Section 2.4, p. 6.
3	**Occupation**	State the interviewer's occupation at the time of the study.	Reported. She was a social work academic and doctoral student. Section 2.4, p. 6.
4	**Gender**	Report the interviewer's gender.	Reported. The interviewer was female. Section 2.4, p. 6.
5	**Experience and training**	Describe the interviewer's relevant experience or training.	Reported. She had doctoral-level training in qualitative research. Section 2.4, p. 6.
6	**Relationship established**	Clarify whether a relationship existed before study commencement.	Reported. The interviewer had no prior relationship with participants. Section 2.4, p. 6.
7	**Participant knowledge of interviewer**	Describe what participants knew about the interviewer and the research.	Reported. Participants were informed about her professional role, research interests, and the purpose of the doctoral study. Section 2.4, p. 6.
8	**Interviewer characteristics**	Report relevant assumptions, interests, or reflexive characteristics.	Reported. The first author's social work background and interest in women's mental health were treated reflexively as part of the analysis. Section 2.5, p. 7.

Domain 2 Study design			
9	**Methodological orientation and theory**	State the methodological and theoretical orientation underpinning the study.	Reported. The study used a critical-feminist epistemological orientation, supplementary qualitative secondary analysis, and reflexive thematic analysis. Sections 2.1 and 2.5, pp. 4 and 6–7.
10	**Sampling**	Explain how participants were selected.	Reported. The mother-only subgroup was purposively selected for the focused research question; all eight mothers in the 15-participant parent dataset were included. Section 2.2, p. 5.
11	**Method of approach**	Describe how potential participants were approached.	Reported. Treating psychiatrists made the initial approach; the first author then provided study information and obtained consent. Section 2.4, p. 6.
12	**Sample size**	Report the number of participants.	Reported. Eight mothers were included. Abstract, p. 1; Section 2.2, p. 5.
13	**Non-participation**	Report refusals or withdrawals and, where available, their reasons.	Partly reported. Refusal and dropout numbers for the parent study are not stated. For the secondary analysis, all eight mothers in the dataset were included and no eligible case was omitted. Section 2.2, p. 5.
14	**Setting of data collection**	State where data collection took place.	Reported. Interviews were conducted face-to-face in a private room at the hospital. Section 2.4, p. 6.
15	**Presence of non-participants**	State whether anyone else was present during data collection.	Reported. No one other than the participant and interviewer was present. Section 2.4, p. 6.
16	**Description of sample**	Provide relevant demographic, clinical, or contextual characteristics.	Reported. Clinical characteristics and eligibility are described in Section 2.2; sociodemographic and family characteristics appear in Table 1. pp. 5 and 24.
17	**Interview guide**	Describe the interview guide, prompts, and any piloting or review.	Reported. A literature-informed semi-structured guide and metaphor prompt were used. The guide was reviewed by multidisciplinary experts and five women, leading to merged questions and simplified wording. Sections 2.3 and 2.6, pp. 6 and 8; full guide in Supplementary Material 2.
18	**Repeat interviews**	State whether repeat interviews were conducted and how many.	No repeat interviews were conducted; each participant was interviewed once. Section 2.4, p. 6

19	**Audio or visual recording**	State whether interviews were recorded.	Reported. Interviews were audio-recorded with consent. Section 2.4, p. 6.
20	**Field notes**	State whether field notes were made during or after interviews.	Reported. Field notes were prepared for each interview. Section 2.4, p. 6.
21	**Duration**	Report the approximate duration of interviews or focus groups.	Reported. Interviews lasted approximately 70 minutes. Sections 2.2 and 2.4, pp. 5-6.
22	**Data saturation**	Explain whether and how saturation was considered.	Reported. Saturation was not used because this was a closed secondary dataset; adequacy was considered through information power. Section 2.2, p. 5.
23	**Transcripts returned**	State whether transcripts were returned to participants for comment or correction.	Transcripts were not returned to participants for comment or correction. Section 2.4, p. 6

**Domain 3 Analysis and findings**			
24	**Number of data coders**	State how many researchers coded the data.	Reported. The first author coded and led the analysis; co-authors served as critical dialogue partners rather than independent consensus coders. Sections 2.5-2.6, pp. 7-8.
25	**Description of the coding tree**	Describe the organization of codes and themes.	Reported. Code comparison and theme development are described in Section 2.5, and Table 2 presents the thematic structure and underlying codes. pp. 7 and 24-25.
26	**Derivation of themes**	Clarify whether themes were pre-specified or developed from the data.	Reported. Initial coding was inductive; feminist social work and intersectionality were sensitizing perspectives rather than a predetermined coding framework. Section 2.5, p. 7.
27	**Software**	Name any software used to manage or analyze the data.	Reported. MAXQDA 2024 was used to organize data, codes, and extracts, not to generate interpretations. Section 2.5, p. 7.
28	**Participant checking**	State whether participants provided feedback on the findings.	Not conducted for themes or findings. Feedback from five women occurred before data collection and concerned only the interview guide. Section 2.6, p. 8.
29	**Quotations presented**	Use identifiable participant quotations to support findings.	Reported. Quotations are linked to participant codes throughout the Results and in Tables 2 and 3. pp. 8-13 and 24-25.
30	**Data and findings consistent**	Show a clear relationship between the presented data and interpretations.	Reported. Each theme is supported by extracts followed by analysis; Table 2 links themes, codes, participant coverage, and representative extracts. pp. 8-13 and 24-25.

31	**Clarity of major themes**	Present the principal themes clearly.	Reported. Six themes are introduced, separately headed, and summarized in Table 2. pp. 8-13 and 24-25.
32	**Clarity of minor themes and diverse cases**	Describe variation, contradictions, minor themes, or deviant cases.	Reported. Cross-case variation, contradictions, and qualifying or deviant cases are described; no separate minor-theme level was used. Sections 2.5, 3.6, and 4, pp. 7, 13-15.

Source: Tong A, Sainsbury P, Craig J. Consolidated criteria for reporting qualitative research (COREQ): a 32-item checklist for interviews and focus groups. International Journal for Quality in Health Care. 2007;19(6):349-357. https://doi.org/10.1093/intqhc/mzm042
